# Characterization of a Distinct Population of Circulating Human Non-Adherent Endothelial Forming Cells and Their Recruitment via Intercellular Adhesion Molecule-3

**DOI:** 10.1371/journal.pone.0046996

**Published:** 2012-11-07

**Authors:** Sarah L. Appleby, Michaelia P. Cockshell, Jyotsna B. Pippal, Emma J. Thompson, Jeffrey M. Barrett, Katie Tooley, Shaundeep Sen, Wai Yan Sun, Randall Grose, Ian Nicholson, Vitalina Levina, Ira Cooke, Gert Talbo, Angel F. Lopez, Claudine S. Bonder

**Affiliations:** 1 Centre for Cancer Biology, South Australian Pathology, Adelaide, South Australia, Australia; 2 Co-operative Research Centre for Biomarker Translation, La Trobe University, Melbourne, Victoria, Australia; 3 School of Medicine, University of Adelaide, Adelaide, Australia; 4 Leukocyte Biology Laboratory, Women's and Children's Health Research Institute, Adelaide, South Australia, Australia; 5 La Trobe Institute for Molecular Science, La Trobe University, Melbourne, Victoria, Australia; Brigham and Women's Hospital, United States of America

## Abstract

Circulating vascular progenitor cells contribute to the pathological vasculogenesis of cancer whilst on the other hand offer much promise in therapeutic revascularization in post-occlusion intervention in cardiovascular disease. However, their characterization has been hampered by the many variables to produce them as well as their described phenotypic and functional heterogeneity. Herein we have isolated, enriched for and then characterized a human umbilical cord blood derived CD133^+^ population of non-adherent endothelial forming cells (naEFCs) which expressed the hematopoietic progenitor cell markers (CD133, CD34, CD117, CD90 and CD38) together with mature endothelial cell markers (VEGFR2, CD144 and CD31). These cells also expressed low levels of CD45 but did not express the lymphoid markers (CD3, CD4, CD8) or myeloid markers (CD11b and CD14) which distinguishes them from ‘early’ endothelial progenitor cells (EPCs). Functional studies demonstrated that these naEFCs (i) bound *Ulex europaeus* lectin, (ii) demonstrated acetylated-low density lipoprotein uptake, (iii) increased vascular cell adhesion molecule (VCAM-1) surface expression in response to tumor necrosis factor and (iv) in co-culture with mature endothelial cells increased the number of tubes, tubule branching and loops in a 3-dimensional in vitro matrix. More importantly, naEFCs placed in vivo generated new lumen containing vasculature lined by CD144 expressing human endothelial cells (ECs). Extensive genomic and proteomic analyses of the naEFCs showed that intercellular adhesion molecule (ICAM)-3 is expressed on their cell surface but not on mature endothelial cells. Furthermore, functional analysis demonstrated that ICAM-3 mediated the rolling and adhesive events of the naEFCs under shear stress. We suggest that the distinct population of naEFCs identified and characterized here represents a new valuable therapeutic target to control aberrant vasculogenesis.

## Introduction

The identification of progenitor cells in adult peripheral blood has significant clinical implications for the treatment of multiple diseases. Particular emphasis has been placed on the research and development of vascular progenitor cells with pro-angiogenic potential for wound healing [1], limb ischemia [2], myocardial ischemia [3], [4] as well as the increased vascularisation associated with tumor development, sensitivity to chemotherapy and cancer progression [5], [6], [7], [8]. In addition, the balance between normal and pathological states for cardiovascular disease and diabetes has been linked to the number of circulating endothelial progenitor cells (EPCs) [9], [10], [11], [12]. Despite the exact contribution of EPCs in vasculogenesis still being under intense debate [10], [13], [14], [15], [16], the ability of human EPCs to rescue diminished blood flow in preclinical animal models [17], [18] provided rationales to initiate clinical trials. The results of these studies have found infusion of CD34^+^ and CD133^+^ EPCs to be safe and beneficial in certain circumstances, though the effects in humans have been less robust and much more variable than in preclinical rodent studies [10]. With continued promise of modulating both overzealous and suboptimal vasculogenesis in disease, clearly these cells warrant further investigation.

Despite the absence of a definitive EPC marker and the ambiguous terminology used to define EPCs, the functional distinction between different groups of EPCs (eg ‘early EPCs’ and ‘late outgrowth EPCs’ (also known as endothelial colony forming cells (ECFCs)) is becoming clearer and has been extensively discussed in recent reviews by Yoder, Ingram [15], Dimmeler [19] and Hagensen [20]. Briefly, it is becoming increasingly evident that simple phenotyping for the surface expression of CD34 and VEGFR2 as well as uptake of acetylated-low density lipoprotein (Ac-LDL) and lectin binding are not adequate descriptors of ‘true’ EPCs rather it is the capacity to incorporate into an endothelial lining and conduct endothelial cell functions which are the strict criteria required and can not be obtained outside the living organism [15], [19], [21], [22]. The inclusion of CD133 as a marker of EPCs by Peichev and colleagues provided the first opportunity to distinguish EPCs from CD34^+^VEGFR2^+^ ECs [23]. Interestingly, our current understanding of EPC biology has been largely restricted to ECFCs which are derived from ∼3 week in vitro cell culture and do not express CD133 which suggests a more mature phenotype likely compromised by extensive cell culture [15], [24]. This is critical, as it was the immature EPCs which first demonstrated an ability to contribute to the formation of arteries, capillaries and veins [25] and it is the non-adherent cells in circulation that would be the first responders to a site of vascular injury. To this end, Asahara's laboratory recently demonstrated in an EPC clonogenic assay that a single human umbilical cord blood (HUCB) derived CD133^+^ cell can develop into a colony forming EPC as well as a hematopoeitic progenitor cell [26]. It is our contention that isolating and extensively characterising cells which meet the essential criteria of (i) surface expression of progenitor cell and endothelial cell markers and (ii) functional assays which validate that postnatal endothelial differentiation capacity in vitro and in vivo; will be an essential prerequisite to discovering meaningful surface biomarkers with which the clinical promise of vasculogenesis may ultimately be realised.

Herein, we have isolated cells from HUCB-derived non-adherent CD133^+^ progenitor cell population, enriched for EPCs over 4 days and compared their genomic and proteomic profile against donor matched human umbilical vein endothelial cells (HUVEC). Flow cytometry confirmed an EPC phenotype with surface expression of CD133, CD117, CD34, VEGFR2 and CD31 (PECAM-1) but not lymphocyte, myeloid and platelet surface markers (CD3, CD4, CD8, CD11b, CD14, CD19, CD20 and CD41a). Functional studies also supported an endothelial phenotype as these cells demonstrated binding to *Ulex europaeus* lectin (UEA-1), uptake of acetylated-low density lipoprotein (Ac-LDL), enhancement of 3-dimensional tubes, tubule branching and loops in vitro and upregulation of vascular cellular adhesion molecule (VCAM)-1 following tumor necrosis factor-α (TNFα) stimulation.

Although most studies suggest that the cells gained by the short-term culture assays (eg ‘early’ EPCs) predominantly enhance vessel formation via a paracrine mechanism rather than directly contributing to tube formation [27], [28] we demonstrate in vivo that our cells display the postnatal vasculogenic capability of maturation into an endothelial cell and lining of tubule structure in vivo. Moreover, with an intention to increase the clarity and rigour of the use of the name ‘EPC’, we have coined the term ‘non-adherent endothelial forming cells’ (naEFCs) to define these cells as it highlights their capabilities (endothelial forming) as well as their physical properties (non-adherent).

Genomic profiling identified the increased expression of intercellular cell adhesion molecule (ICAM)-3 on naEFCs when compared to donor matched HUVEC. Tandem mass spectrometry using a periodate oxidation-hydrazide resin capture approach for N-glycosylated surface proteins and flow cytometry confirmed the surface expression of ICAM-3 on the naEFCs but not HUVEC. Importantly, flow cytometry also confirmed the surface expression of ICAM-3 on freshly isolated peripheral blood derived CD133**^+^**CD117**^+^** progenitor cells. Functional analysis demonstrated that ICAM-3 mediated naEFC rolling and adhesion events under shear stress in vitro. Collectively, our studies recognise ICAM-3 as a new adhesive molecule by which circulating naEFCs may contribute to the aberrant vasculogenesis during disease.

## Materials and Methods

### Ethics statement

The collection of primary human umbilical vein endothelial cells (HUVEC) and peripheral blood mononuclear cells (PBMC) for use in this study was given ethical clearance from the Royal Adelaide Hospital (RAH), Adelaide, South Australia. The collection of primary human umbilical cord blood (HUCB) for use in this study was given ethical clearance from the Human Research Ethics Committee of the Children, Youth and Women's Health Service (CYWHS), North Adelaide, South Australia and informed written consent was obtained from all subjects in accordance with the ‘Declaration of Helsinki’. Animal experiments (detailed below) were approved by the Animal Ethics Committee of SA Pathology and conform to the guidelines established by the ‘Australian Code of Practice for the Care and Use of Animals for Scientific Purposes’.

### Isolation and culture of HUCB CD133^+^ non-adherent endothelial forming cells (naEFCs) and early EPCs

HUCB (20–130 ml) was collected from healthy pregnant women undergoing elective caesarean section into MacoPharma cord blood collection bags (MSC1201DU; MacoPharma, Mouvaux, France) prior to dilution 1∶1 with sterile phosphate buffered saline (PBS) and mononuclear cells (MNCs) isolated via Lymphoprep™ (Axis-Shield, Oslo, Norway). MNCs were incubated with 100 µl of human FcR blocking reagent (Miltenyi Biotec, Bergisch Gladbach, Germany) and 100 µl of CD133^+^ microbeads (MACS, Miltenyi Biotec) for 30 minutes at 4°C prior to isolation using the AutoMacsPro (Miltenyi Biotec) as per manufacturer's instructions. CD133^+^ cells were resuspended at a concentration of 0.5–1×10^6^ cells/ml in endothelial growth media (EGM-2, Lonza, Basel, Switzerland) complete with Bullet kit and supplemented with 10% FCS, VEGF (5 ng/ml; Sigma, St Louis, MO, USA), insulin-like growth factor-1 (IGF-1; 0.005 ng/ml; Gibco Invitrogen), basic fibroblast growth factor (bFGF; 1 ng/ml; R&D, Minneapolis, MN, USA) and ascorbic acid (0.1 mM; Sigma). Cells were seeded onto pre-coated fibronectin (50 µg/ml, Roche, IN, USA) wells in a falcon 24-well plates (BD Biosciences) and incubated at 37°C and 5% CO_2_ in a Hera Cell incubator (Thermo Scientific, Waltham, MA, USA). Whilst in culture the non-adherent cells were transferred to a new pre-coated fibronectin well with fresh EGM-2 media (plus supplements) every 48–72 h. Unless otherwise stated, naEFCs were cultured for 4–5 days prior to harvesting for further analysis.

As comparator cells, ‘early EPCs’ were obtained by HUCB-derived MNC CD133^+^ isolation using magnetic beads and cultured, as above, on fibronectin in EGM-2 for 5 days as an adherent population of cells which adopted a spindle-shaped cluster similar to the first morphological description of ‘early EPCs’ [22].

### Peripheral blood MNCs and human umbilical vein endothelial cells (HUVEC)

Peripheral blood MNCs (PBMNCs) were isolated from healthy individuals collected in lithium heparin coated Vacuette tubes (Greiner Bio-One, Kremsmuenster, Austria). Primary HUVEC were extracted from human umbilical veins by collagenase digestion and cultured in HUVE media (Media 199 (Sigma); containing 20% FCS (Hyclone, Utah, USA), endothelial growth factor supplement (BD BioSciences, North Ryde, NSW, Aust.), 1.5% sodium bicarbonate, 2% HEPES buffer solution, Penicillin Streptomycin, sodium pyruvate (Gibco Invitrogen, Gaithersburg, MD, USA), heparin and non-essential amino acids (Sigma)) as previously described [29], [30] and were used no later than two passages.

### Flow cytometric analysis of cell surface protein expression

Cells (naEFCs, early EPCs, freshly isolated CD133^+^ cells, HUVEC and PBMNCs) were analysed for cell surface expression of various markers by flow cytometry. Where indicated, cells were treated with 5 ng/ml TNFα (R&D Systems) for 24 hours prior to harvest. Cells were treated with 10 µl Human FcR block diluted in 30 µl HUVE wash (Media 199 (Sigma), 2% FCS, 1% 10 mM HEPES and 1% penicillin streptomycin solution (Gibco) prior to addition of primary antibodies. Cells were incubated in 100 µl HUVE wash with either mouse anti-human VEGFR2 (1 µg, Santa Cruz Biotechnology, Inc, Santa Cruz, CA, USA), mouse anti-human vascular cell adhesion molecule-1 (VCAM-1, 2 µg, generated in-house, clone 61.10F12), mouse anti-human ICAM-3 (1 µg, BD Biosciences) or relevant isotype control (1 µg, BD Biosciences) followed by 1 µg biotinlyated goat anti-mouse Ig in 100 µl HUVE wash. Cells were blocked with 5 µl normal mouse serum (SA Pathology, Adelaide, Australia) then immediately incubated with conjugated streptavidin (PE, APC or PE-Cy7) (BD Biosciences) added at 0.2 µg per test along with panels of mouse anti-human conjugated antibodies; anti-CD117-APC or PerCP-Cy5.5, anti-CD11b-pacific blue, anti-CD14-APC, anti-CD144-FITC or PE, anti-CD31-PE or V450, anti-CD34-Percp-Cy5.5, anti-CD45-FITC or Amcyan, anti-CD41a-FITC, anti-CD3-FITC, anti-CD4-PE, anti-CD8-FITC, anti-CD19-APC, anti-CD20-PE, anti-CD10-APC, anti-CD90-APC, anti-CD38-PE-Cy7 or FITC (all BD Biosciences) and anti-CD133-PE (Miltenyi Biotec) used as per manufacturer's instructions for flow cytometry in a final volume of 80 µl of HUVE wash. Cells were resuspended in FACS fix (1% formaldehyde, 20 g/L glucose, 5 mM sodium azide in PBS) prior to analysis using a FACS Aria II (BD Biosciences) with FACS DIVA (BD Biosciences). Further analysis was performed using FCS Express 4 Flow Cytometry: Research Edition (De Novo Software, CA, USA).

### Incorporation of Acetylated-low density lipoprotein (Ac-LDL) with Ulex europaeus lectin (UEA-1) binding

Cells were incubated at 37°C for 4 hours with 10 µg/mL 1,1′-dioctadecyl-3,3,3′,3′-tetramethylindocarbocyanine (DiI) perchlorate-Ac-LDL (DiI-Ac-LDL; Biomedical Technologies, Stoughton, MA, USA) and 10 µg/mL UEA-1-FITC (Sigma). The percentage of cells within each population that incorporated DiI-Ac-LDL and bound UEA-1-FITC was assessed using flow cytometry.

### Matrigel tube formation assay – in vitro

In vitro tube formation of HUVEC and naEFCs was assessed using a Matrigel matrix. HUVEC were stained with 10 µg/ml DiI-Ac-LDL (Biomedical Technologies, Stoughton, MA) for 4 hours at 37°C, 5% CO_2_, washed once and incubated overnight at 37°C, 5% CO_2_; naEFCs were stained with 0.5 µM CFDA-SE (CFSE, Invitrogen) in 0.1% FCS in PBS for 10 minutes prior to washing. The next day, 12 µl Matrigel (BD Biosciences) was added to wells in a pre-warmed ibiTreat Angiogenesis μ-slide (Ibidi, Martinsried, Germany) and incubated at 37°C for ≥30 minutes. Labelled HUVEC and naEFCs were seeded together in Matrigel at a cell density of 1.7×10^4^ HUVEC alone or with 0.7×10^4^ naEFCs per well, in duplicate. Tube formation was monitored regularly and 10–15 overlapping phase contrast images were captured using an inverted IX70 microscope 4×/0.13NA obj, an S15 F view camera and Analysis Life Sciences software (Olympus) after 6 hours. These overlapping images were “stitched” together using PTGui Pro software (New House Internet Services B.V., Rotterdam, The Netherlands) and tube, branch and loop numbers were quantified from each entire well using the WimTube algorithm (Wimasis GmbH, Munich, Germany) [31]. Fluorescent and phase contrast images were also captured using an IX81 microscope (Olympus) with 10×/0.4NA obj and a Hamamatsu Orca-ER camera. Fluorescence images were acquired using CellR software (Olympus Soft Imaging System).

### Matrigel plug assay – in vivo

Female NOD/SCID mice (Animal Resources Centre, Western Australia) were used between 6–8 weeks of age and housed in specific pathogen-free conditions in the SA Pathology Animal Care Facility. naEFCs were stained with 0.5 µM CFSE in 0.2% FCS in PBS for 7 minutes prior to 5×10^5^ naEFCs being mixed in 500 µl Matrigel with 2 µg/ml basic fibroblast growth factor (bFGF, R&D) and 50 units/ml heparin (Sigma-Aldrich) and injected subcutaneously into one flank. Each mouse also received a control plug of 500 µl Matrigel with bFGF and heparin without cells in the alternate flank. Mice were humanely killed by cervical dislocation 7 days post Matrigel injection. Plugs were removed, washed in PBS and frozen in O.C.T. compound (Tissue-Tech, Tokyo, Japan) prior to 10 µM sections being cut, acetone fixed and stained for CD144 (1∶500, Sigma) for 2 hours at RT prior to being washed and incubated with Alexa594-conjugated anti-goat (1∶500 dilution, Invitrogen) for 30 min on ice and mounted with Prolong Gold antifade reagent with DAPI (Invitrogen). Negative controls were secondary antibody alone. Images were produced using Nikon C1-Z Confocal Microscope at the Detmold Imaging Core Facility, SA Pathology where the C1-Z was equipped with three solid lasers, (Sapphire 488 nm, Compass 532 nm, and Compass 405 nm near UV) and an inverted Nikon E-2000 Fluorescence Microscope. The objective used was a Nikon Plan Apo-chrome 60× water (NA = 1.2) and the triple labelled samples were imaged with three separate channels (PMT tubes).

To determine functionality of tubes formed in Matrigel plugs mice were injected i.v. with 200 µg of tetramethylrhodamine isothiocyanate (TRITC)-labeled lectin (*Ulex europeaus*, Sigma) 7 days post Matrigel injection. After 20 min of circulation, mice were heart-perfused with PBS followed by 4% paraformaldehyde (PFA) in PBS. Plugs were removed, washed in PBS and frozen in O.C.T. compound. Four µm frozen sections were analyzed. For 2-photon microscopy a LSM 710 NSO microscope (Carl Zeiss Pty Ltd, Jena, Germany) with plan-Neofluar objective 20×/0.8 (∼3× digital magnification) and laser lines of 488 nm wavelength for CFSE, 555 nm wavelength for TRITC-lectin and 405 nm wavelength for DAPI detection was used. Images were processed by Zen system 2011 (Carl Zeiss) and PHOTOSHOP CS4 (Adobe Systems, San Jose, CA).

### Dispase digestion of Matrigel plugs

Using the aforementioned protocol of Matrigel plug assay, plugs were harvested from NOD/SCID mice 7 days post CFSE-naEFC and Matrigel injection and washed in PBS. Plugs were macerated and incubated in 3 ml of Dispase (BD Biosciences) for 2 hours at RT to isolate the cells within the excised plug. The mixture was then pipetted repetitively to disperse the cells and 0.01M EDTA/PBS was added to stop the Dispase activity. Cells were put through a 70 µm cell strainer (BD Biosciences) to remove debris before flow cytometry staining as described above in ‘Flow cytometric analysis of cell surface protein expression’.

### Hematopoietic colony formation assay

Freshly isolated CD133^+^, CD133^−^ cells as well as naEFCs were plated in methylcellulose with a 10× cell solution (200 µl, IMDM with 2% FCS) mixed with 2 ml of MethoCult (Stem Cell Technologies) containing erythropoietin (EPO; 3 U/ml), granulocyte macrophage colony-stimulating factor (GM-CSF; 20 ng/ml), stem cell factor (SCF; 50 ng/ml) and interleukin 3 (IL-3, 10 ng/ml). The cell/MethoCult mix (0.37 ml) was added to 24 well plate wells at 1670 cells/well. Assays were incubated at 37°C, 5% CO_2_ and hematopoietic colonies were scored after 14 days. Cytospins were prepared from hematopoietic colonies and stained by May Grunwald/Giemsa stain (both BDH).

### Cytokine and Adhesion bead assays

The secretion of cytokines and adhesion molecules by naEFCs into day 4 culture supernatant was assessed using the FlowCytomix Human Th1/Th2 11 plex Kit and the FlowCytomix Human Adhesion 6 plex Kit (both Bender MedSystems, GmbH Campus, Vienna, Austria) as per the manufacturer's instructions and analysed using the FC500 (Beckman Coulter) and FlowCytomix Pro (Bender MedSystems).

### Protein expression analysis by mass spectrometry

naEFCs and adherent HUVEC were oxidized by adding 1 mM of sodium periodate (Sigma) in PBS at 4°C for 10 minutes. After removal of sodium periodate with PBS the cells were lysed for 15 minutes (100 mM CH_3_COONa, pH 5.5, 1% Glucoside, 1% Triton X100) and the oxidised glycoproteins were conjugated to the hydrazide-linked beads at room temperature for at least 24 hours. Non specifically bound proteins were removed by several washes with 6 M guanidine HCl in 50 mM Tris pH 8.0. Proteins coupled to the beads were reduced with 0.01 M DTT in 50 mM Tris, pH 8.0 at 60°C for 1 hour followed by alkylation with 5 times molar excess of Iodoacetamide for 30 minutes in the dark at room temperature. After several washes the proteins were digested for 2 hours with 1 µg of trypsin in 60 µl of 50 mM Tris. pH 8.0, 10% ACN. The released tryptic peptides were removed by further washes. Residual trypsin was inactivated by reduction and alkylation as described above followed by a wash with 6 M Guanidine HCl, 50 mM tris, pH 8.0 buffer. N-linked glycopeptides were released from the beads by addition of 500 Units of PNGase F and incubated overnight at 35°C. The released peptides were dried and resuspended in 1 µl of 8M urea and diluted with 0.1% formic acid(aq). The peptides were analysed by LC-MALDI-tof/tof mass spectrometry or LC-MicroTOF-Q-MS/MS (Bruker Daltonics, Bremen, Germany).

For MALDI-MS, tryptic peptides were separated by RP-HPLC (Ultimate 3000 system, Dionex, USA) using capillary column (Vydac Everest, C_18_ 300 Å, 5 µm, 150 µm ID, 150 mm length). Samples were washed for 10 minutes with buffer A (5% (v/v) ACN and 0.05% (v/v) TFA(aq)) at 1 µL/min. Peptides were eluted with a gradient of 1% increment/minute of buffer B (80% (v/v) ACN in 0.05% TFA(aq)) for 12 minutes followed by 1.5% increment/minute of buffer B for 25 minutes. The column eluate was spotted onto a Anchorchip 800/384 plate (Bruker Daltonics, Germany) with a sheath flow of CCA (α-cyano-4-hydroxycinnamic acid) matrix using Proteineer fc fraction collector (Bruker Daltonics) with 10 seconds for each spotted fraction. Each spot was analysed using a UltraflexIII MALDI-tof/tof-MS (Bruker Daltonics) under control of Bruker's proprietary software, WARP-LC.

For LC-MicroTOF-Q-MS, tryptic peptides were separated by RP-HPLC (RSLCnano system, Dionex, USA) using a nano column (Acclaim Pepmap RSLC, 2 µm C_18_ 75 µm×150 mm) following trapping on a trap column (Acclaim Pepmap 100 nanotrap C18 100 µm×20 mm) prior to injection into the MicroTOF-Q MS instrument. The MS-instrument was setup for the highest sensitivity and the data collected were analyses by Bruker Data Analysis software package.

All MS and MS/MS data were searched using three search engines: Mascot (Matrixscience.co.uk), X!Tandem (The Global Proteome Machine) and OMSSA (NCBI). Initial search results from all three search engines were then statistically analysed at the peptide level using Peptide Prophet [32]. Peptide Prophet results were then analysed using iProphet [33], which combines evidence from multiple identifications of the same peptide across multiple search engines and spectra, and finally with Protein Prophet [34] to assess confidence in identifications at the protein level. Only identifications satisfying a 1% false discovery rate were accepted.

### Isolation of total RNA from naEFCs and HUVEC

Total RNA was isolated from naEFCs and HUVEC using RNEasy micro plus or RNEasy mini kits (QIAGEN, Hilden, Germany). RNA integrity and quantity was determined using Agilent 2100 bioanalyzer.

### Gene expression analysis by microarray

150 ng of RNA from naEFCs or donor matched HUVEC was amplified and labelled using Applause™ WT-AmpST/WT-Amp Plus ST Systems. (NuGen Technologies Inc., San Carlos, CA, USA). The labelled and amplified RNA was hybridized to Genechip Affymetrix Human Exon 1.0ST arrays as per the manufacturer's protocol (Affymetrix, Santa Clara, CA, USA) in the microarray facility at Mater Adult Hospital, Brisbane, Australia. Human Affymetrix exon arrays were scanned with Gene chip 3000 7G scanner. Robust multi-array analysis (RMA) was applied for normalizing and summarizing probe level intensity measurements from Affymetrix gene chips. Hybridization quality for each array was assessed using box plots and principal component analysis (PCA) of probe-level data. A parametric Welch's t-test (where variances were not assumed equal) was performed on 19524 probes for naEFCs with a p-value cut off of 0.05 and a fold change cut off of 1.5. Multiple significant probes for the same were removed and the probe with the highest fold change retained for further analysis. Multiple testing correction (Benjamini and Hochberg False Discovery Rate) was then applied to genes that had passed the parametric Welch's t-test based on the total detected probe-set of 14246 probes to reduce false positives. Significantly upregulated genes were grouped according to their potential relevant functions in progenitor cells. Functional categorization of genes was performed using a combination of Agilent technologies gene ontology classifications and Ingenuity Pathway Analysis (IPA). The data discussed in this manuscript have been deposited in the NCBI gene expression omnibus (GEO Accession number: GSE25979).

### Quantitative Polymerase Chain Reaction (qPCR)

Quantification of mRNA levels was carried out using qPCR. Primers designed for human ICAM-3 (F-5′AGTGACGACGGACGCAGCTT3′, R-5′GGGCATGTGGCTCGGTCAAT3′) using Primer Blast (NIH, MD, USA), and purchased from GeneWorks (Hindmarsh, SA, Aust.). Where possible, primers were designed to span an intron/exon border to ensure no genomic DNA amplification. qPCR amplification was performed using QuantiTect™ SYBR Green master mix (QIAGEN) on a Rotor-Gene thermocycler (Corbett Research, Mortlake, NSW, Aus.) with reaction parameters: 15 minutes at 95°C, then cycling of 10 seconds 95°C, 20 seconds 55°C and 30 seconds 72°C; for 45 cycles followed by a melt phase. Data obtained was analysed using Rotor-Gene Analysis Software version 6 (Corbett Research). Relative gene expression levels were calculated using standard curves generated by serial dilutions of cDNAs normalised to the human house-keeping gene cyclophillin A (CycA) (F-5′GGCAAATGCTGGACCCAACACAAA-3′, R-5′CTAGGCATGGGAGGGAACAAGGAA3′).

### Parallel Plate Flow Chamber Assay

HUVEC were cultured until confluent on μSlideIV^0.4^ chambers (Ibidi) and treated with or without 5 ng/mL TNFα for 5 hours. naEFCs or heparinized whole blood at 1∶10 dilution with Hanks Balanced Salt Solution (HBSS, Sigma) were treated with or without antibodies to ICAM-3 (1 µg, BD Biosciences) or isotype control (1 µg, IgG2b, BD Biosciences) 30 minutes prior to cell perfusion at 0.8–1×10^6^ cells/ml across substratum by syringe pump (NE-1000, New Era Pump System, Inc, Wartagh, NY, USA) at constant rate of 2 dynes/cm^2^ for 5 minutes followed by HBSS wash. Notably, due to the low proportion of naEFCs in HUCB (<3% of cells) [35], the number of cells (0.5–2×10^6^ cells at day 4) available was limited so donors were pooled immediately prior to the experiments. Cells were visualised using 10×/0.3 NA objectives and phase-contrast microscopy on an inverted microscope and images recorded using a digital camera (Olympus IX70 and SIS F-view, Olympus, Mount Waverly, Vic, Aus). Five random areas per slide recorded for analysis using AnalySIS Life Sciences software (Olympus). Adherent cells were defined as those remaining stationary for at least 10 seconds.

### Statistical Analysis

Results were expressed as mean ± standard error of the mean (SEM) from at least 3 experiments. Unless otherwise stated, an unpaired Student T-test, 1- or 2-way ANOVA for multiple comparisons was performed to determine statistical significance between groups with p values<0.05 considered significant.

## Results

### Enrichment of a non-adherent CD133^+^ with an endothelial progenitor cell phenotype

MNCs were isolated from HUCB via lymphoprep density gradient centrifugation prior to enrichment for the progenitor cell marker CD133 cells using magnetic sorting. CD133**^+^** enriched MNCs were cultured in EPC specific growth media on fibronectin-coated wells at a concentration of 0.5–1×10^6^ cells/ml. At day 2 of culture, the majority of cells remained non-adherent and were replated into a new fibronectin-coated well with fresh media for continued culture.

**Figure 1:**
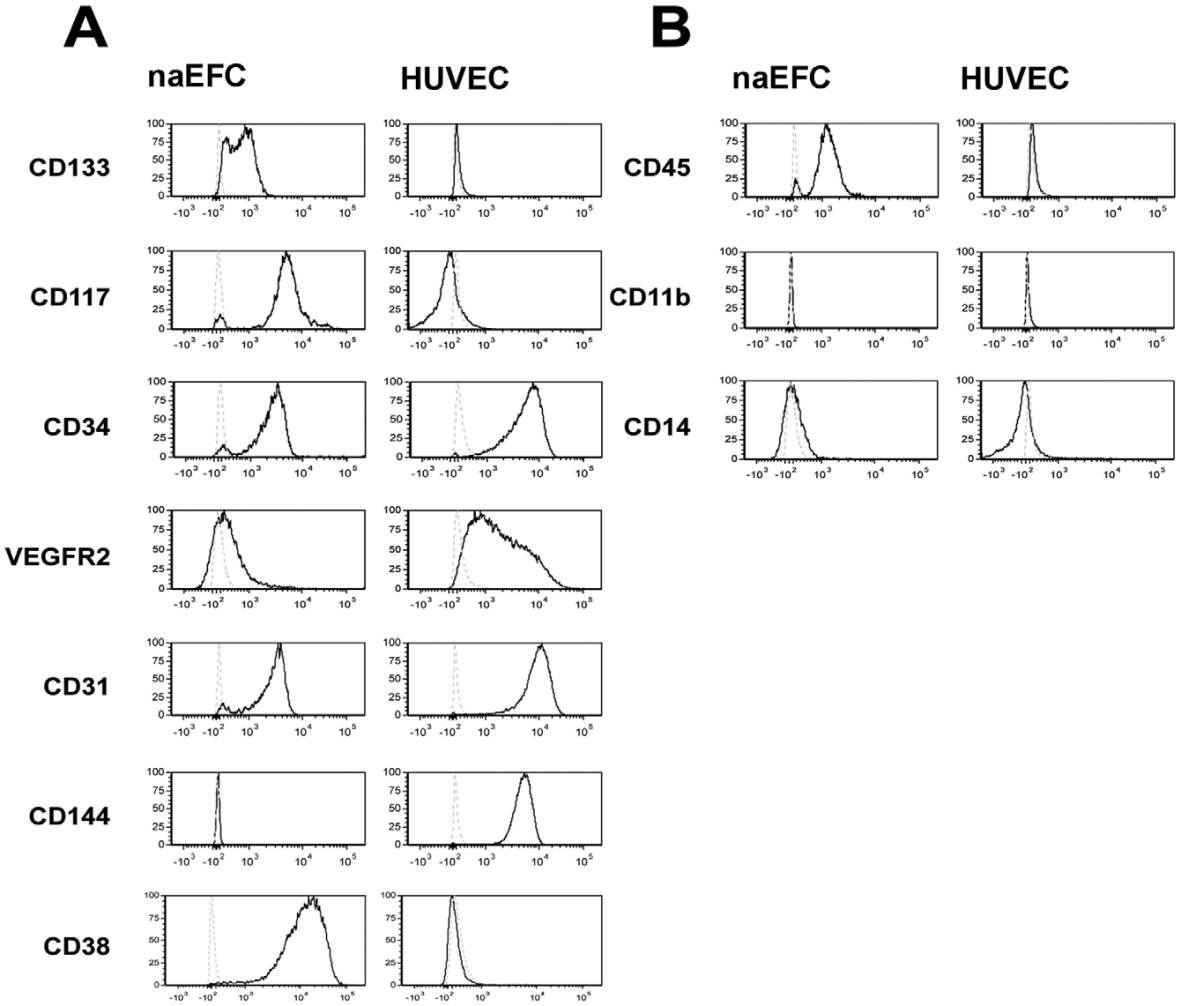
Surface expression profiling of freshly isolated CD133^+^ cells, naEFCs and HUVEC. In (A), freshly isolated CD133^+^ cells were phenotyped for hematopoietic progenitor cell and endothelial cell markers by flow cytometry. In the histograms, the light dotted lines represent unstained cells and the dark lines represent stained cells of one representative experiment from n≥3. In (B), CD133^+^ enriched cells at 4 days of culture (naEFCs) and HUVEC were more extensively assessed for surface antigen phenotype. The histograms show one representative experiment from n≥3 with the light and dark lines as above. In (C), the pan-leukocyte marker CD45 and the myeloid markers CD11b and CD14 were examined with the light dotted lines representing unstained cells and the dark lines representing stained cells of one representative experiment from n≥3.

Phenotyping of cell surface markers on the HUCB-derived naEFC population was performed after 4 days of culture by flow cytometry and compared to mature ECs (HUVEC) isolated from the same donor. Investigation of the hematopoetic progenitor cell markers CD133, CD117 and CD34 profile [15] on the naEFCs suggests that the percentage of cells expressing CD133 is sustained at approximately 69±5% at day 4 of culture (Figure 1A). Also shown in Figure 1A, CD117 was expressed on 80±5% of the naEFCs at day 4 of culture. The third progenitor marker, CD34 demonstrated a similar profile with approximately 78±4% of the naEFCs expressing CD34 at day 4 of cell culture (Figure 1A). As expected, CD133 and CD117 expression was significantly lower on HUVEC (2±1% and 10±5%, respectively) while CD34 exhibited robust expression of 82±9% on these cells (Figure 1A). We next investigated the mature endothelial cell markers of VEGFR2, CD31 and CD144. Also shown in Figure 1A, CD31 was expressed on 86±2% of the 4 day cultured naEFCs and was similar to the 98±1% expression by HUVEC. The number of naEFCs expressing VEGFR2 was uniformly lower in the naEFCs at 15±2% when compared to the HUVEC population at 45±10%. Notably, the need for a commercially available reliable antibody to VEGFR2 is still missing [36] and as such the data presented herein may under represent the true levels of VEGFR2 expressed by these cells. CD144 was not detected by flow cytometry on the naEFCs, but was expressed at high levels on 99±2% of the HUVEC population (Figure 1A). These data suggest that 4 days of culture allows for enrichment of a non-adherent EPC population which express both hematopoietic progenitor cell and endothelial cell markers are the subject of further investigation in this study.

As circulating EPCs can be subdivided into two main categories, hematopoietic lineage and non-hematopoietic lineage [15], [28], [37], we investigated the naEFCs for surface expression of CD45, CD11b and CD14. Flow cytometric analysis suggested that CD45 was expressed by a majority of the naEFCs with little to no CD45 expression observed on the HUVEC population (Figure 1B). Notably, the ratio of mean fluorescence intensity (MFI) of CD45 expression over isotype control staining on the naEFCs was significantly lower than that expressed by CD14^+^ monocytes from freshly isolated PBMNCs (ratio MFI naEFCs 21±6 versus monocytes 48±2, n = 3). Also shown in Figure 1B, we were unable to detect both CD11b and CD14 on both the naEFCs and HUVEC. This is in contrast to the 100% expression by the monocyte population gated by forward and side scatter profile from PBMNCs (not shown).

To investigate whether the culture conditions may have contributed to the naEFC phenotype we compared these cells to freshly isolated CD133^+^ cells. As shown in Figure S1, CD133^+^ isolated cells which have not been subjected to a 4 day enrichment process exhibit a mostly uniform expression of CD133, CD117 and CD31, but a heterogeneous expression of CD34, CD144, CD45, CD11b and CD14. These data support previous reports of CD133 being expressed on a variety of cell types [38]. To confirm that the naEFCs are different from other ‘early EPCs’ we compared the non-adherent naEFCs to the established protocol of adherent CD133^+^ cells cultured in EGM-2 for 5 days [15], [19], [22]. As shown in Figure S1, the ‘early EPCs’ expressed a very different surface antigen profile with little to no CD117, CD34 and CD144 but a homogeneous expression of CD31, CD45, CD11b and CD14. Taken together, these data suggest that we have enriched for a non-adherent EPC-like cell within 4 days of CD133^+^ cell isolation and that these cells differ from both the heterogeneous population of freshly isolated CD133 cells as well as ‘early EPCs’ [15], [19].

A recent study by Prokopi et al suggested that MNCs may acquire ‘endothelial’ characteristics of CD31 expression by taking up platelet microparticles [39]. To confirm that expression of CD31 is not platelet microparticle derived, we investigated the surface expression of the platelet-specific marker CD41a on naEFCs. As shown in Figure S2A, the CD133**^+^**CD117**^+^** double positive cells (left panel) did not express CD41a at the cell surface (right panel) and as such are not platelet microparticle derived.

As CD45 is considered to be a pan-leukocyte marker typically expressed by hematopoietic cells, its moderate expression on the naEFCs warranted investigation of additional lineage markers. As shown in Figure S2B, the naEFCs did not express CD3, CD4, CD8, CD20, they showed low expression of CD19 (8±4% positive) and significant expression of CD90 (63±5% positive) and CD38 (84±3% positive). Taken together, these results suggest that the heterogeneous nature of CD133**^+^** HUCB derived MNCs enriches into an ‘EPC-like’ progenitor population by day 4 of culture.

### Functional characterization of naEFCs

**Figure 2:**
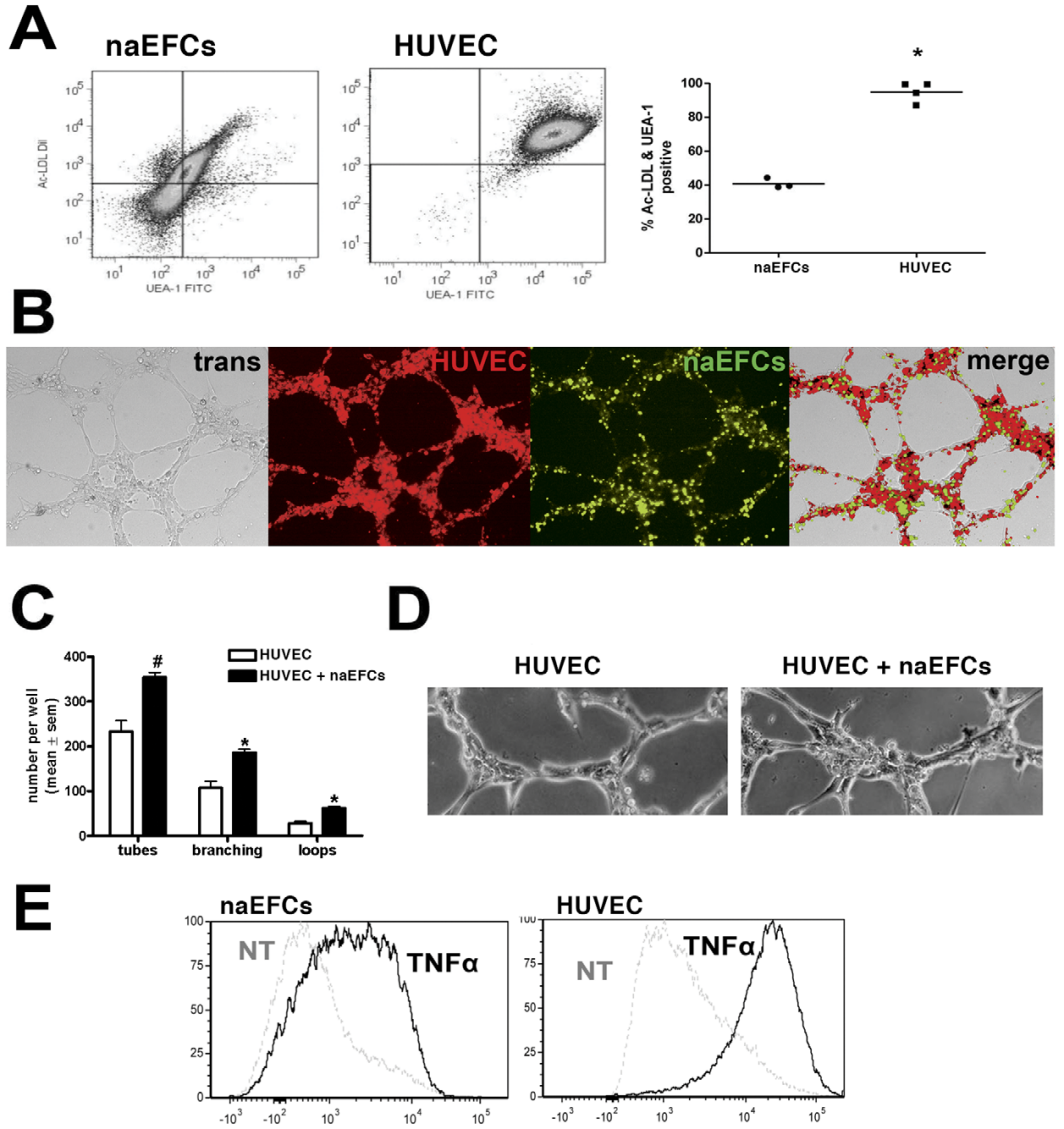
Vascular properties of naEFCs. In (A), representative dot plots from one experiment show the incorporation of DiI-Ac-LDL and binding of UEA-1-FITC by naEFCs and HUVEC. The percentage of cells double positive for DiI-Ac-LDL uptake and binding of UEA-1-FITC was quantified. *p<0.05, versus naEFCs, n≥3. In (B), representative image of a Matrigel assay at 6 hours post seeding of CFSE-labeled naEFCs (green) and DiI-Ac-LDL positive HUVEC (red). Results represent one experiment of n = 5 with images captured by transmission and confocal microscopy. In (C), the number of tubes, branches and loops formed in the endothelial tube formation Matrigel assay in vitro with HUVEC alone or HUVEC co-cultured with naEFCs. #*p* = 0.05 versus HUVEC alone, *p<0.05 versus HUVEC alone, n = 5. In (D), a representative image of the tube formation of HUVEC alone (upper image) or HUVEC co-cultured with naEFCs (lower image) in vitro. Results represent one experiment of n = 5 with images captured by transmission microscopy. In (E), representative histograms showing increased surface expression of VCAM-1 following TNFα administration for 24 hours on naEFCs (left panel) and HUVEC (right panel), n = 4–6.

The naEFCs were next scrutinized for their functional capabilities. First, we examined their capability to take up acetylated low-density lipoprotein (Ac-LDL) and bind *Ulex europeaus* lectin (UEA-1). As shown in Figure 2A, flow cytometric analysis suggests that the progenitors take up Ac-LDL and bind UEA-1 with the single cluster supporting a homogeneous population. Importantly, the number of these cells double positive for Ac-LDL uptake and lectin binding is statistically lower than donor matched HUVEC, this is indicative of an immature vascular phenotype [26], [40].

To begin to explore a pro-angiogenic potential of the naEFCs we investigated their secretion of cytokines and soluble adhesion molecules. Using the Human Th1/Th2 11 plex and Human Adhesion 6 plex FlowCytomix bead arrays we detected high levels of interleukin (IL)-8 (1008±380 pg/ml from 10^6^ cells) and soluble CD31 (49±9 pg/ml from 10^6^ cells) in the culture supernatant of the untreated naEFCs when compared to media alone.

We next tested the naEFCs in endothelial tube formation assays using Matrigel; an extracellular matrix derived from murine sarcoma cells that supports vascular tube formation in vitro and thus mimics in vivo vasculogenesis. In an in vitro assay, while the naEFCs did not form tubules on their own (not shown), when co-cultured with HUVEC, naEFCs demonstrated an ability to migrate to each other as well as the HUVEC to interact and form a capillary-like network within 6 hours of seeding. This is shown in Figure 2B where DiI-Ac-LDL and CFSE were used in a dual labelling system to distinguish HUVEC and naEFCs, respectively. As shown in Figure 2C, quantification of the angiogenic effect of the naEFCs in vitro was executed using the WimTube algorithm [31] and identified a significant increase in (i) the number of tubes formed, (ii) the number of branching points extruding from the tubes (exemplified in Fig. 2D) and (iii) the number of loops formed.

Expression of the endothelial cell predominant adhesion molecule vascular cell adhesion molecule (VCAM)-1 was assessed on naEFCs and HUVEC following stimulation with TNFα by flow cytometry. As expected, HUVEC exhibited increased levels of cell surface VCAM-1 following TNFα treatment for 5 hours. However, this was not observed on the naEFCs (not shown). Suarez et al performed a TNFα exposure time-course on HUVEC wherein VCAM-1 expression was significantly elevated at 24 hours compared to 5 hours [41]. We thus increased the TNFα exposure to 24 hours for both the naEFCs and HUVEC and observed that surface expression of VCAM-1 was significantly increased on both the naEFCs and HUVEC populations when compared to untreated controls (Figure 2E).

**Figure 3:**
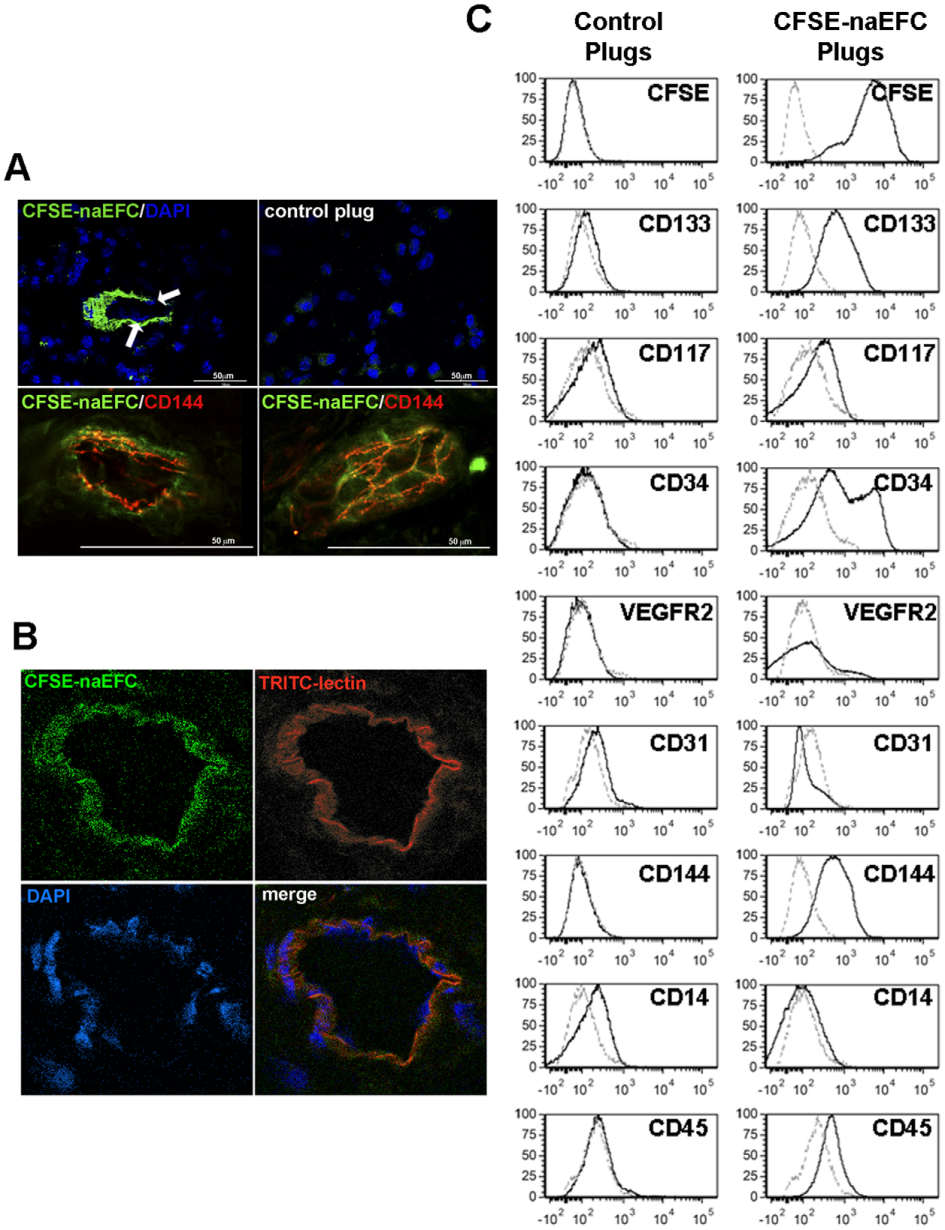
naEFCs express mature EC markers and form perfused tubes in vivo. In (A), CFSE-labelled naEFCs mixed with Matrigel prior to injection into the flank of NOD/SCID mice, after 7 days the plugs were removed, processed and sections counterstained for nuclei with DAPI prior to imaging by confocal microscopy. The upper left image shows the cross section of a CFSE-naEFC generated tube-like structure (green) within which the nuclei of cells can be seen (blue) at 60× mag (arrows). The upper right image is the control plug in which no naEFCs were added. Images represent one experiment of n≥3. Similar sections were stained for CD144 and images captured by confocal microscopy with CFSE-naEFCs (green) exhibiting CD144 (red) as a cross section of a tube (lower left image) and CD144 staining in the junctions of the CFSE-naEFCs (lower right panel). Images are a representative of n≥3. In (B), similar experiments were executed and at day 7 post-implant the mice were injected i.v. with TRITC-lectin prior to exsanguinations, plugs removed, processed and sections counterstained for nuclei with DAPI prior to imaging by confocal microscopy. The representative image shows the cross section of a CFSE-naEFC generated tube-like structure (green, upper left image), TRITIC-lectin (red, upper right image), DAPI counterstain (blue, lower left image) and the merged image (lower right). In (C), CFSE-naEFCs were digested from explanted Matrigel plugs using dispase and phenotyped for hematopoietic progenitor cell and endothelial cell markers by flow cytometry (right panel); cells from contra-lateral control Matrigel plugs were similarly examined for antigen expression (left panel). In the histograms, the light dotted lines represent unstained cells and the dark lines represent stained cells of a representative of repeated experiments.

An in vivo Matrigel plug assay supports the generation of lumen containing vasculature lined by mature human ECs. As shown in Figure 3A, when 5×10^5^ CFSE-labelled naEFCs were mixed with 500 µl Matrigel (without co-culture with HUVEC) and injected subcutaneously into the flank of NOD/SCID mice, within 7 days the green fluorescent naEFCs generated lumen, forming vasculature within the plug. This is shown using confocal microscopy with the naEFCs (green) and a counterstain for nuclei (blue) from frozen sections wherein nuclei can be identified within the circular structure formed by the CFSE-naEFCs (Figure 3A, arrows). Figure 3A also shows CD144 expression (red) on a cross-section of a CFSE-naEFC formed vessel (left image) as well as intercellular expression of CD144 on CFSE-naEFCs (right image). Although, the anti-CD144 antibody used here is cross-reactive with both human and mouse CD144, with the increased intensity of CD144 staining at the exact location of the green CFSE-naEFC it is tempting to speculate that the naEFCs have differentiated into a mature CD144^+^ EC in vivo, a marker which we could not readily detect on the in vitro cultured naEFCs (Figure 1B).

Further investigation of tube forming capabilities was undertaken by i.v. injection of with 200 µg of tetramethylrhodamine isothiocyanate (TRITC)-labeled UEA lectin and after 20 min of circulation, mice were heart-perfused with PBS followed by 4% paraformaldehyde (PFA) in PBS. Matrigel plugs were explanted, postfixed and sections analysed by 2-photon microscopy. As shown in Figure 3B, CFSE-naEFCs (green) formed a tubule structure within which the circulating TRITC-lectin bound (red) suggesting lumen formation by the human cells.

To determine the homogeneity of the naEFCs following 7 days in vivo, we executed flow cytometric analysis on dispase digested cells from the explanted Matrigel plugs. As shown in Figure 3C, mononuclear cells isolated from the naEFC containing plugs, but not the contra-lateral control plugs (which contained no naEFCs), held CFSE expressing cells. Gating on the CFSE positive cells and using human specific monoclonal antibodies revealed their high expression of CD133, CD34 and CD144, low expression of CD117 and VEGFR2, and no expression of CD31. As important, these cells were CD45 low and did not express CD14 (Figure 3C, right panel histograms). As expected, surface expression of the aforementioned human antigens was not detectable on the MNC gated cells digested out of the control plugs (Figure 3C, left panel histograms).

### Multipotency of naEFCs

**Figure 4:**
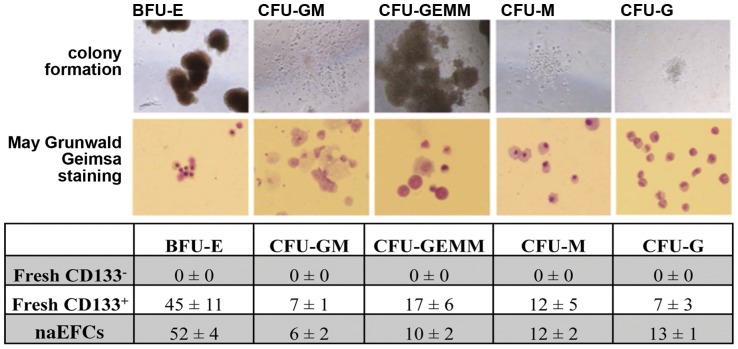
Hematopoietic properties of naEFCs. naEFCs were seeded in MethoCult and growth factors GM-CSF, IL-3, SCF and EPO for 14 days prior to colony counting and staining with May Grunwald/Giemsa to assess cellular morphology. naEFCs formed blast-forming unit-erythroid (BFU-E), colony-forming units (CFU)-GEMM, -GM, -G and -M colonies in methylcellulose. Colony formation was photographed and quantified after 14 days and compared between naEFCs and freshly isolated CD133^+^ and CD133^−^ cells (mean ± sem, n = 3).

Asahara recently demonstrated that a single HUCB derived CD133^+^ cell could give rise to cells of either the hematopoietic or endothelial lineage [26]. Using a similar methylcellulose hematopoietic colony formation assay we executed a fate analysis of naEFCs. As shown in Figure 4, when the naEFCs were cultured with GM-CSF, SCF, IL-3 and EPO for 14 days a variety of cell types were detected including early progenitors of both erythrocyte and myeloid lineages (CFU-GEMM), the erythrocyte lineage (BFU-E), the granulocyte/monocyte progenitor lineages CFU-GM, CFU-G and CFU-M. May Grunwald/Giemsa-stained cytospin preparations of methylcellulose cultured cells confirmed the presence of hematopoietic cells, including those of the myeloid lineage (Figure 4). Also shown in Figure 4, a comparison of freshly isolated HUCB derived CD133^+^ and CD133^−^ cell fractions alongside the naEFCs which suggests a similarity between the naEFCs and the CD133^+^ cells with respect to the number and type of colonies formed. These functional assays suggest that the naEFCs have multipotential lineage capabilities and that given the correct growth factor cocktail that a variety of lineages can be obtained.

### Intercellular adhesion molecule (ICAM)-3 mRNA is upregulated in naEFCs

**Figure 5:**
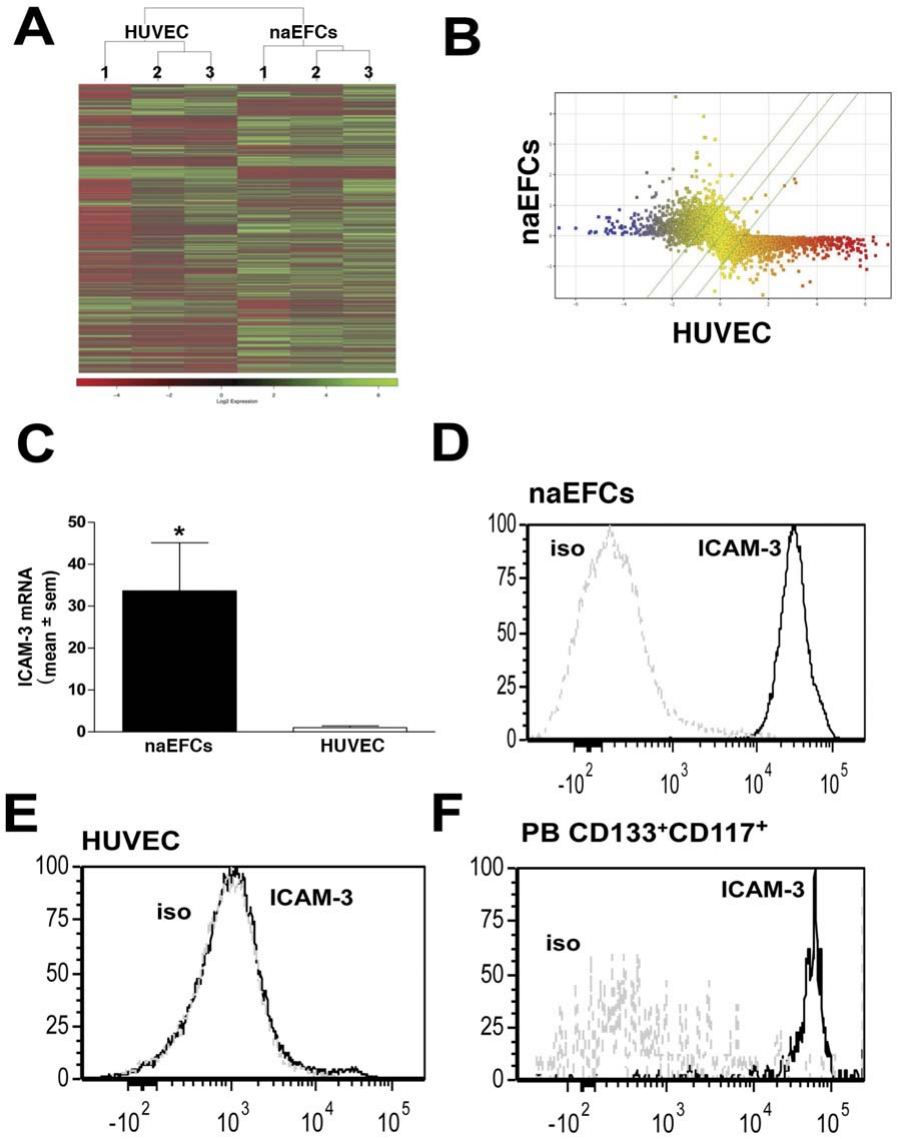
Gene expression analysis of naEFCs versus HUVEC. In (A), a heat map illustrating the hierarchical clustering of Log2 relative gene expression in 3 separate HUVEC and naEFC samples. In (B), scatter data showing the average gene expression data in naEFCs and HUVEC. The dots represent the gene expression of UCB CD133+ 4 day cultured naEFCs versus HUVEC. The diagonal lines indicate the cut off value of 1.5 fold activation and genes coloured on the basis of expression level (yellow, evenly expressed genes; blue, naEFC upregulated genes; red, naEFC downregulated genes). In (C), ICAM-3 mRNA levels in naEFCs and HUVEC as determined by qPCR with relative gene expression normalised to CycA. Data are expressed as relative fold change (mean ± sem) normalised to HUVEC, n = 3,*p<0.05 versus HUVEC. In (D–F), flow cytometric analysis of ICAM-3 on (D) naEFCs, (E) HUVEC and (F) freshly isolated peripheral blood CD133^+^CD117^+^ gated cells. Light dotted line represents the unstained control and the dark line represents cells stained for ICAM-3. One representative experiment is shown n≥3.

Gene expression profiling of naEFCs versus their donor matched HUVEC was performed using Genechip Affymetrix Human 1.0 ST Exon arrays (Figure 5A). Figure 5B illustrates the gene expression scatter profile after robust multi-array analysis (RMA) normalization and the complete microarray data set is available via the NCBI Gene Expression Omnibus (GEO) (Accession number GSE25979). Using a *p*-value cut-off of ≤0.05 (see methods for full statistical analysis) and a fold change cut-off of 1.5, a total of 977 had a significantly higher expression and 1128 genes had a significantly lower expression in naEFCs when compared to HUVEC. Notably, data analysis confirmed a higher expression of progenitor cell markers CD133, CD117, CD44 and chemokine (C-X-C motif) receptor 4 (CXCR4) in the naEFC population when compared to HUVEC (Table 1). Similarly, mRNA expression of the mature endothelial cell markers (CD31, CD144, CD62E and von Willebrand Factor) was significantly higher in HUVEC when compared to the naEFCs (Table 1).

**Table 1:** Fold change values of well-established markers in the naEFCs when compared with HUVEC.

Gene Symbol	Gene Name	naEFCs
CD133	prominin-1	8.8 ↑
CD117	proto-oncogene c-Kit	5.9 ↑
CD44	CD44	5.7 ↑
CXCR4	Chemokine (C-X-C motif) receptor 4	6.2 ↑
CD31	PECAM-1	2.1 ↓
CDH5	VE-cadherin	25.0 ↓
CD62E	E-selectin	20.7 ↓
vWF	von Willebrand Factor	16.0 ↓
CD34	CD34	No change
eNOS	nitric oxide synthase 3	1.4 ↓
VEGFR2	vascular endothelial growth factor receptor 2	12.9 ↓
ICAM-3	intercellular adhesion molecule 3	4.4 ↑

↑ denotes significantly higher mRNA expression;

↓ denotes significantly lower mRNA expression.

We next investigated the potential relevant function of the genes upregulated in the naEFCs using a combination of Gene spring GX11 Gene Ontology classifications, ingenuity pathway analysis (IPA) and Ace view search engines. The most significantly represented biological processes include cancer, cardiovascular diseases, cardiovascular system development and function, cell signaling, cellular growth and proliferation, haematological disease, cellular function and maintenance, lipid metabolism and metabolic disease. Importantly, all of these processes are known to be associated with or involved in vascular dysfunction [42].

Using the aforementioned search engines, we next mined the data for genes which may contribute to the functional activity of circulating naEFCs. Within this search, ICAM-3 was identified as a new putative adhesion molecule for naEFCs, exhibiting a 4.3 fold increase in mRNA expression over their donor matched HUVEC (Table 1). To validate these results new biological replicates were examined by qRT-PCR with results demonstrating ∼30-fold increase in mRNA expression of ICAM-3 in progenitors versus donor matched HUVEC (Figure 4C).

### Mass spectrometry confirms ICAM-3 surface expression on naEFCs

Proteome profiling using tandem mass spectrometry is an established technology for the detection of cell surface proteins. Protein glycosylation is a common post-transcriptional modification with N-linked glycosylation prevalent in proteins destined for the extracellular environments [43]. Herein we enriched for surface glycoproteins on the naEFCs and donor matched HUVEC by selective capture of N-glysocylated membrane proteins onto beads using hydrazide chemistry followed by enzymatic release of the peptides and subsequent MALDI-tof/tof-MS and MicroTOF-Q-MS/MS tandem mass spectrometry. Protein hit lists were generated by searching Swissprot database. The integrity of the hits was ensured by the inclusion of N-glycosylations sites as well as an increment of the mass of 1 Da due to the change of Asn to Asp. Multiple MS runs using up to 3 biological replicates identified over 750 glycoproteins from the naEFC preparations with recognition of CD133, CD117 and CD44 on these cells but not HUVEC (Table 2); CD31 was detected on both cell types and CD144 was detected on HUVEC but not the naEFCs. Within this list, ICAM-3 was repeatedly detected on the naEFCs but was not observed in the HUVEC glycoprotein preparations. As shown in Figures 5D and 5E, flow cytometric analysis confirmed the surface expression of ICAM-3 on the naEFCs and not on HUVEC. Confirmation of ICAM-3 expression on bona fide circulating progenitor cells was obtained using flow cytometry which repeatedly demonstrated its expression on freshly isolated CD133^+^CD117^+^ progenitor cells circulating in the peripheral blood (Figure 5F).

**Table 2:** MALDI-tof/tof-MS of N-glysocylated naEFC membrane proteins using hydrazide-bead capture.

Gene symbol	Protein name	Sequences of identified glycopeptides
PROM1_HUMAN	Prominin-1 (CD133)	VLNSIGSDID**NVT**QR
KIT_HUMAN	Proto-oncogene c-kit (CD117)	SLYGKED**NDT**LVR
CD44_HUMAN	CD44-antigen (CD44)	AF**NST**LPTMAQMEK
PECA1_HUMAN	Platelet endothelial cell adhesion molecule (CD31)	L**NLS**CSIPGAPPANFTIQK
CDH5_HUMAN	Vascular endothelial cadherin (CD144)	**NTS**LPHHVGKIK
ICAM3_HUMAN	Intercellular adhesion molecule-3 (ICAM-3)	EIVC**NVT**LGGER

The consensus motif for N-linked glycosylation is highlighted in bold.

### ICAM-3 mediates naEFC cell rolling and adhesion events under shear stress

**Figure 6:**
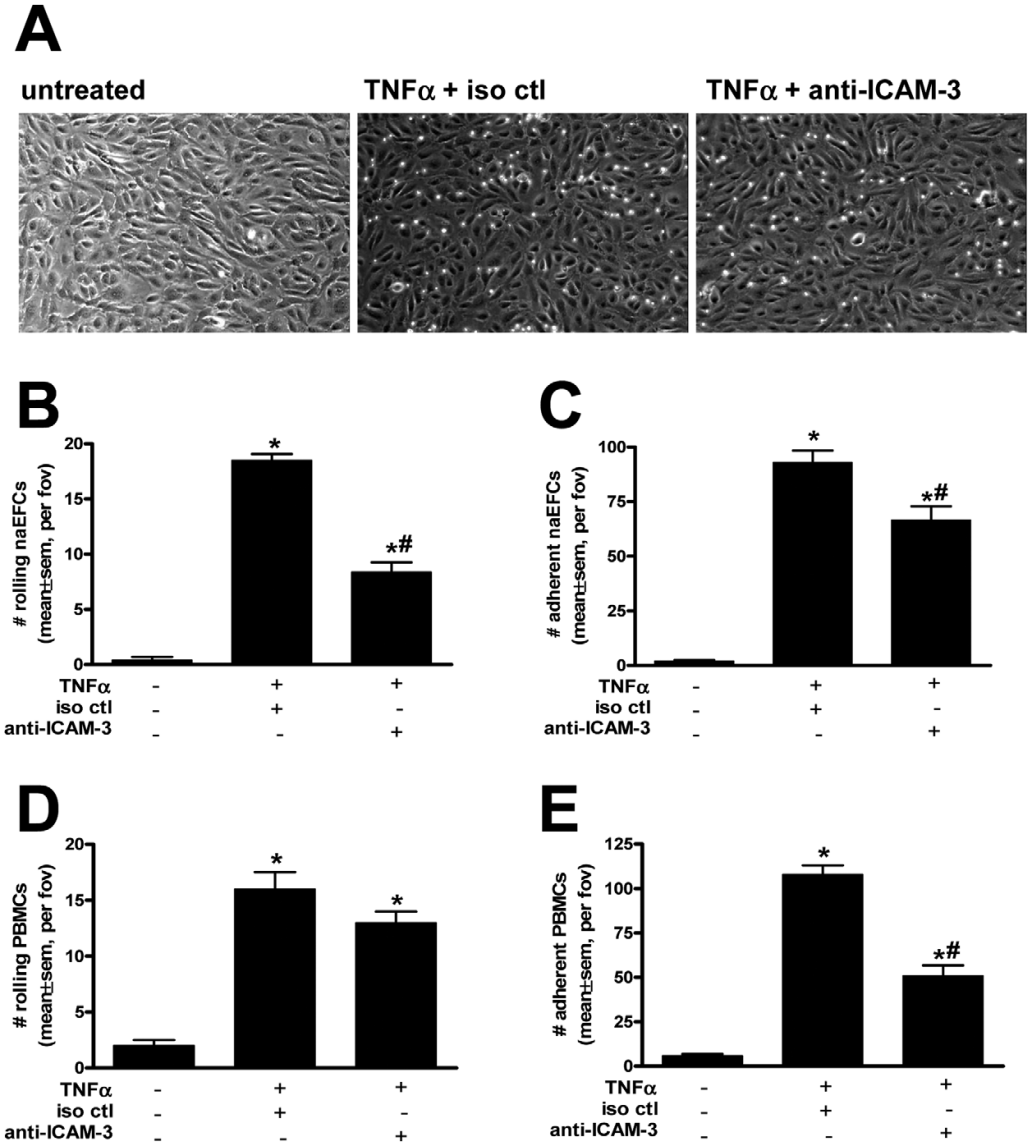
ICAM-3 mediates rolling and adhesion of naEFCs. In (A), still images of Video S1 illustrate the interaction of naEFCs with untreated (left panel), TNFα treated (5 ng/ml for 5 hours, middle and right panels) where naEFCs were pre-treated with an isotype control antibody (middle panel) or an antibody to ICAM-3 (right panel) prior to perfusion over HUVEC at 2 dynes/cm^2^. In (B and C), data of rolling and adherent naEFCs is represented as the mean ± sem per field of view (fov) for n = 3;*p<0.05 versus untreated; #*p*<0.05 versus iso ctl. In (D and E), data of rolling and adherent whole blood treated with an isotype control or antibody to ICAM-3 is represented as the mean ± sem per field of view (fov) for n = 4–5;*p<0.05 versus untreated; #*p*<0.05 versus iso ctl.

To investigate the potential use of ICAM-3 by naEFCs, a parallel plate flow chamber assay was performed. As shown in Figure 6A–C and Video S1, when naEFCs were perfused over untreated HUVEC at a rate of 2 dynes/cm^2^, negligible naEFC rolling or adhesion was observed. By contrast, when the HUVEC were stimulated with TNFα for 5 hours a significant increase in naEFC rolling and adhesion was observed (Figures 6A–C and Video S1). When naEFCs were pre-treated with a blocking ICAM-3 antibody 30 minutes prior to perfusion over TNFα activated HUVEC a significant decrease in the number of rolling and adherent naEFCs was observed when compared to both isotype control and untreated cells (Figure 6A–C and Video S1).

Interestingly, studies investigating the role of ICAM-3 in whole blood rolling and adhesion on TNFα-treated HUVEC suggested a different role for this adhesion molecule. As shown in Figures 6D–E, when compared to an isotype control antibody, the administration of a blocking antibody to ICAM-3 did not affect the rolling of whole blood cells on the endothelium but demonstrated a significant attenuation in cell adhesion.

Based on these results we returned to the Matrigel assay and investigated the role of ICAM-3 in tubule formation using progenitor cell co-culture experiments with HUVEC. In repeated experiments we were unable to demonstrate that administration of a blocking antibody to ICAM-3 altered tube formation (not shown).

## Discussion

Regulation of blood vessel homeostasis and turnover is essential for the function of all organs and tissues during embryonic development and in adulthood. Thus, there is great interest in understanding the mechanisms that regulate endothelial cells in different vessels as well as identifying possible sources for their replacement when vascular complications occur. A seminal article identified EPCs as cells that circulate in peripheral blood and express CD34 and VEGFR2 [17], however mature EC and hematopoietic cells also express these cellular markers and they can be mobilised to sites of neovascularisation. A decade and a half later, a definitive delineation of EPCs and protocols to unambiguously isolate these cells in vivo remains elusive. In fact, a human EPC clonogenic assay recently developed by Asahara's laboratory demonstrated that a single HUCB derived CD133^+^ cell can develop into a colony forming EPC as well as a hematopoeitic progenitor cell. This is congruent with the identification of a CD133^+^CD34^+^CD45^+^CD38^+^ common progenitor for endothelial, myeloid and lymphoid precursors in HUCB [44] and multipotent progenitor cells in human peripheral blood [45]. An important role for CD133 on EC precursors was recently demonstrated by Janic et al who showed that HUCB-derived CD133^+^ cells in culture contained mainly EPCs and that long term in vitro conditions facilitated the maintenance of these cells in the state of commitment towards endothelial lineage [46].

In this study we have extensively characterized a tube-forming non-adherent population of EPCs (naEFCs) and executed a genomic and proteomic comparison between these cells and donor matched HUVEC. Herein we demonstrate that HUCB derived CD133^+^ non-adherent progenitor cells cultured for 4 days in vascular cell supportive media expressed CD133, CD117, CD34, CD31, VEGFR2, CD45, CD90 and CD38 at the cell surface but not CD3, CD4, CD8, CD10, CD11b, CD14, CD19, CD20 or CD41a. These progenitor cells demonstrated multipotent pro-angiogenic potential with colony formation in Methocult, indicative of hematopoietic cell growth, as well as features of endothelial cells with (i) incorporation of Ac-LDL and binding of UEA-1, (ii) the release the pro-angiogenic cytokine IL-8 and soluble adhesion molecule CD31, (iii) upregulation of VCAM-1 when stimulated with TNFα, (iv) when seeded together with HUVEC in Matrigel in an in vitro assay the progenitor cells exhibited vascular potential by aligning with tubular structures, increasing tube number, vascular branching from the tubes as well as loops and (v) a capability to contribute to lumen containing tubule structures in vivo which express CD144. A genomic and proteomic profile comparison between the naEFCs and HUVEC identified ICAM-3 as a previously undescribed biomarker of naEFCs. Functional analysis using adhesion assays under shear flow, identified ICAM-3 as new adhesion molecule for circulating naEFCs.

Gene expression profiling of human EPCs is not new, with nearly a dozen published reports currently available. However, our investigation of non-adherent CD133^+^ human naEFCs in short-term culture is novel with the most closely reported studies including those of Igreja and colleagues, who investigated day 0 and 13 cultured CD34^+^CD133^+^VEGFR2^+^ adherent and non-adherent cells derived from HUCB [47] and Jaatinen and coworkers who compared HUCB derived freshly isolated CD133^+^ sorted versus CD133^−^ cells [48]. A close comparison of their published gene expression profiles with that of our study supports the identification of the adhesion molecule ICAM-3 on freshly isolated and short-term cultured EPCs [47] and as such supports our contention of ICAM-3 as an adhesion molecule for circulating progenitors with vasculogenic potential. Importantly, we have taken the next critical step by using glycoproteomics by mass spectrometry together with flow cytometry to confirm the surface expression of ICAM-3 on the surface of naEFCs as well as freshly isolated CD133^+^CD117^+^ circulating PBMNCs.

Cellular adhesion molecules (CAMs) serve to anchor serum factors and blood cells to the endothelium and play a role in establishing the phenotype or inflammatory and tumor vasculature. Of the 5 identified intercellular CAMs in humans, ICAM-3 is best known for its constitutive expression on human leukocytes and while there is little evidence for its expression on endothelial cells in vitro [49], it has been identified on vessels in benign and malignant tumors [50], [51], [52], [53], [54], [55] while weakly or not at all found on endothelial cells during inflammation [53], [56]. Moreover, ICAM-3 has been identified in early stages of vascular proliferation [53], shown to be inversely correlated with the level of vascular differentiation in infantile hemangiomas [55] and that it contributes to the control of the integrity of human bone marrow endothelial layers, likely via an association with an ERM protein moesin and production of reactive oxygen synthase [57]. Herein, parallel plate flow chamber assays identified a new role for ICAM-3 on naEFCs, namely promoting them to roll and adhere to the TNFα-activated HUVEC. Also in this study we investigated the role of ICAM-3 in tubule formation using Matrigel. In repeated experiments we were unable to demonstrate that administration of a blocking antibody to ICAM-3 attenuated tube formation in vitro and in vivo. This is consistent with endothelial cell adhesion molecules being expressed on the luminal side of the vasculature and as such provides additional information for the function of ICAM-3 on circulating endothelial progenitor cells.

With respect to a binding ligand for ICAM-3, it is well described as the third known ligand for the β_2_-integrin lymphocyte function associated antigen (LFA)-1 [58] and has also shown binding capabilities to CD209 (Dendritic Cell-Specific Intercellular adhesion molecule-3-Grabbing Non-integrin; DC-SIGN). While DC-SIGN is primarily known for establishing the first contact between DCs and resting T cells [59] there is some evidence that it is expressed on a select population of endothelium with one report identifying DC-SIGN on placental vasculature [60]. We were unable to identify CD18 or DC-SIGN on TNFα-activated HUVEC (not shown) which suggests that an additional ligand for ICAM-3 on activated HUVEC is yet to be identified. One report by Oostendorp et al suggests that ICAM-3^+^ bone marrow MNCs can bind to stroma via the integrin α_5_β_1_ (VLA5) [61]. We recently demonstrated that TNFα increases the surface expression of α_5_β_1_ on HUVEC [62], and together with data showing that ligation of integrin α_5_β_1_ promotes tumor angiogenesis [63] and α_5_-null carcinomas exhibit reduced blood vessel formation [64] it is tempting to speculate that ICAM3^+^ naEFCs bind to activated endothelium via α_5_β_1_ to promote vascular development. When we investigated the contribution of ICAM-3 in the rolling and adhesive events of whole blood leukocytes we observed a striking difference to that of the naEFCs inasmuch as blocking ICAM-3 attenuated the adhesion but had no effect on leukocyte rolling. The lack of a direct homologue to ICAM-3 in rodents limits the tools available to easily dissect and characterize the adhesive properties of this molecule in vivo. In mouse and rat genomes, remnants of the *Icam3* gene are detectable; however, in these species it has been inactivated during rodent evolution with numerous mutations such as base substitutions, small indels and retrotransposon insertions identified [65].

## Conclusions

Herein we have isolated, enriched for and then characterized a human umbilical cord blood derived CD133^+^ population of non-adherent endothelial forming cells (naEFCs) which expressed both hematopoietic progenitor cell markers and mature endothelial cell markers. Non-adherent EFCs demonstrated functional capabilities expected of EPCs in vitro and are distinguished from ‘early EPCs’ as they did not express the myeloid markers CD11b or CD14 and demonstrated vessel formation in vivo. Extensive genomic and proteomic analyses of naEFCs showed that ICAM-3 is expressed on their cell surface which may act as an adhesion molecule to mediate their rolling and adhesive events to the vasculature under shear stress. The mechanisms and factors controlling progenitor cell recruitment are not well known and are loosely based on the leukocyte recruitment cascade of events which includes the selectin family (ie P-, E- and L-selectin) for transient interactions to initiate tethering and rolling of circulating cells along the endothelium, integrin binding to vascular and intercellular adhesion molecules (eg VCAM-1) [66], [67]. The percentage of EPCs is <0.01% of the circulating white blood cells, their recruitment to sites for vascularisation must therefore be governed by specific profile of adhesion molecules. With our identification of ICAM-3 on the surface of freshly isolated, and importantly not cultured, circulating CD133^+^CD117^+^ progenitor cells it is our contention that ICAM-3 may contribute to the neovascularisation and warrants further investigation as a new opportunity for diagnostic and/or therapeutic potential.

## Supporting Information

Figure S1 **Surface expression profiling of early EPCs.** Early EPCs were phenotyped for hematopoietic progenitor cell (CD117, CD34), endothelial cell (CD31, CD144) and leukocyte cell (CD45, CD11b, CD14) markers by flow cytometry. In the histograms, the light dotted lines represent unstained cells and the dark lines represent stained cells of one representative experiment from n≥3.
(TIF)

Figure S2 **Surface expression profiling of naEFCs and PBMNCs.** In (A), a representative dot plot of CD133^+^CD117^+^ double positive naEFCs were examined for CD41a surface expression by flow cytometry. The light dotted line represents the unstained control and the dark line represents cells stained for CD41a. One representative experiment is shown from n = 5. In (B), naEFCs and PBMNCs were assessed for the expression of lineage markers CD3, CD4, CD8, CD20, CD19 or CD90. Light dotted line represents the unstained control and the dark line represents cells stained for the surface antigen. One representative experiment is shown from n≥3.
(TIF)

Video S1 **Parallel plate flow chamber video of HUVEC pre-treated without or with TNFα (5 ng/ml, 5 hours) prior to perfusion of naEFCs pre-treated with an isotype control (iso control) antibody or an ICAM-3 blocking antibody (1 µg, 30 min) at 2 dynes/cm^2^ for 5 minutes.** Playback speed is 1×.
(MP4)
