# Supplementary figures and images for: Characterization of a Distinct Population of Circulating Human Non-Adherent Endothelial Forming Cells and Their Recruitment via Intercellular Adhesion Molecule-3
Source: PLoS One. 2012 Nov 7;7(11):e46996. doi: 10.1371/journal.pone.0046996 (PMC3492591; doi:10.1371/journal.pone.0046996)

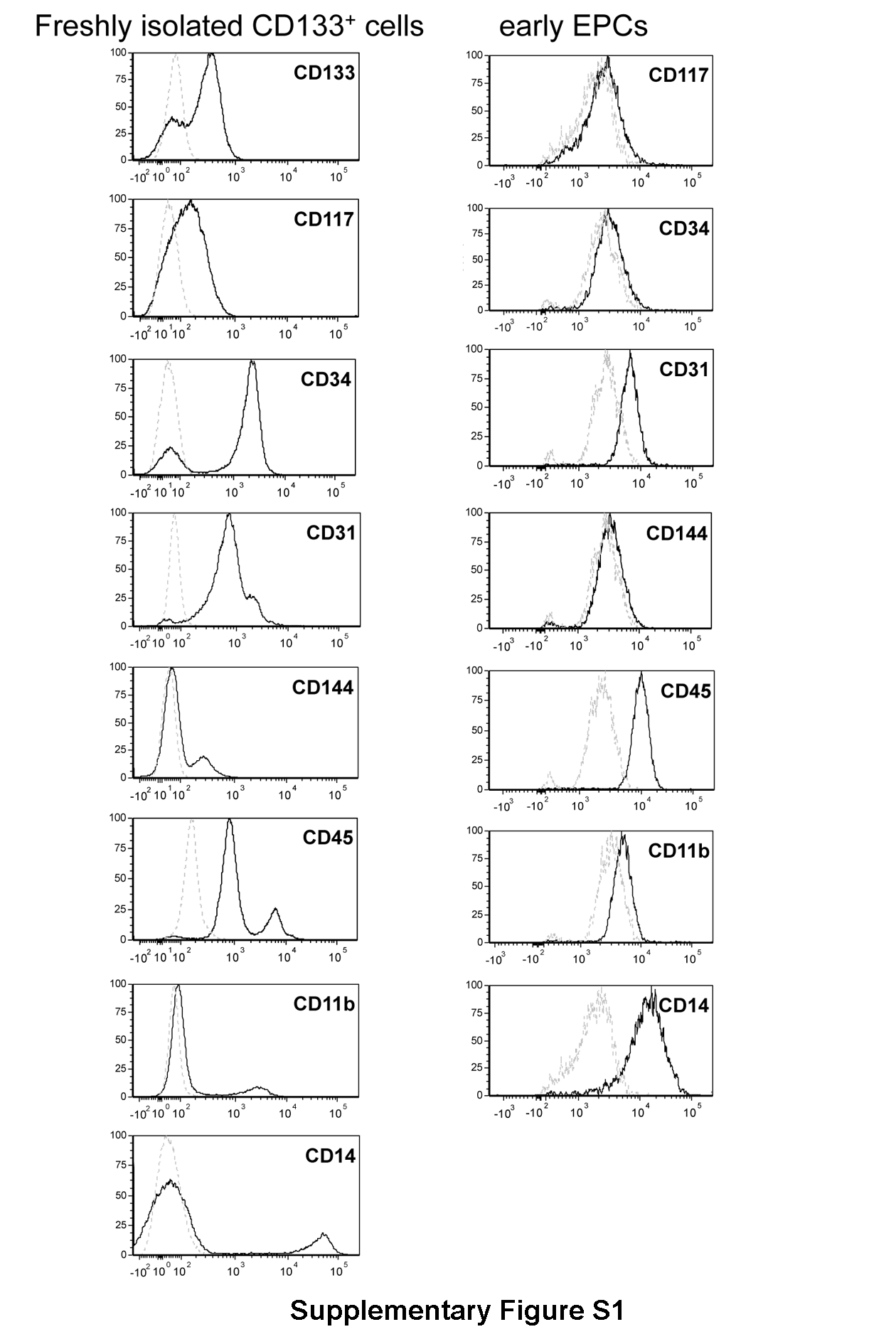

Supplement: Figure S1 — Surface expression profiling of early EPCs. Early EPCs were phenotyped for hematopoietic progenitor cell (CD117, CD34), endothelial cell (CD31, CD144) and leukocyte cell (CD45, CD11b, CD14) markers by flow cytometry. In the histograms, the light dotted lines represent unstained cells and the dark lines represent stained cells of one representative experiment from n≥3. (TIF) [file pone.0046996.s001.tif]

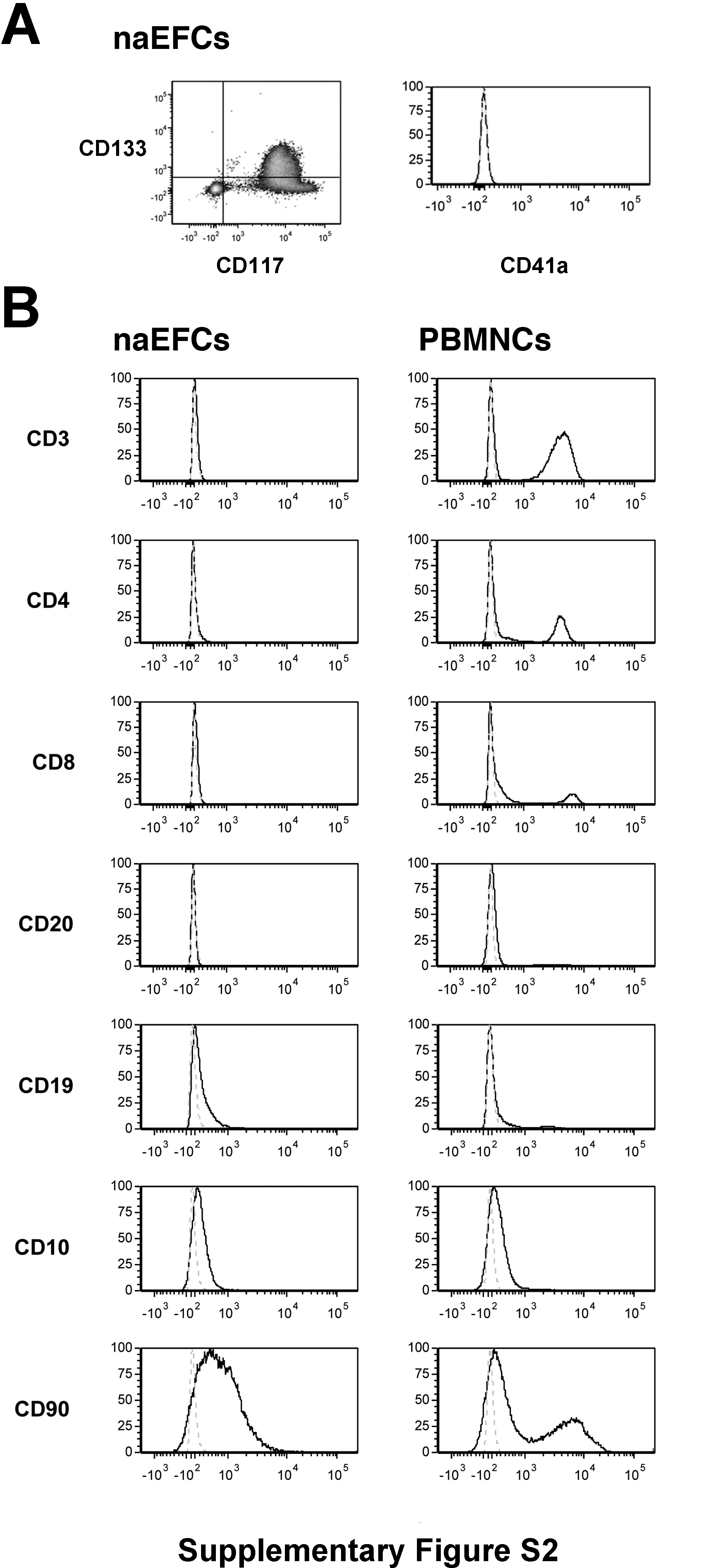

Supplement: Figure S2 — Surface expression profiling of naEFCs and PBMNCs. In (A), a representative dot plot of CD133+CD117+ double positive naEFCs were examined for CD41a surface expression by flow cytometry. The light dotted line represents the unstained control and the dark line represents cells stained for CD41a. One representative experiment is shown from n = 5. In (B), naEFCs and PBMNCs were assessed for the expression of lineage markers CD3, CD4, CD8, CD20, CD19 or CD90. Light dotted line represents the unstained control and the dark line represents cells stained for the surface antigen. One representative experiment is shown from n≥3. (TIF) [file pone.0046996.s002.tif]
